# Long-term effects and quality of life following definitive bile duct reconstruction

**DOI:** 10.1097/MD.0000000000012684

**Published:** 2018-10-12

**Authors:** Włodzimierz Otto, Janusz Sierdziński, Justyna Smaga, Krzysztof Dudek, Krzysztof Zieniewicz

**Affiliations:** a Department of General, Transplant & Liver Surgery; b Department of Medical, Informatics & Telemedicine; c Central Teaching Hospital, Medical University of Warsaw, Poland, Warsaw, Banacha 1a, Poland.

**Keywords:** bile duct injury, bile duct reconstruction, hepaticojejunostomy, quality of life

## Abstract

The study covered a cohort of 236 patients with transection of hepatic duct. It aimed to assess the long-term outcome of the reconstruction and a patient's quality of life.

The literature contains many controversies over timing of biliary reconstruction and who ought to repair the injury but just few reports on the long-term outcomes and patient's quality of life.

The bile duct system was reconstructed by hepaticojejunostomy in 236 patients. Of these, 139 patients were initially repaired at a public hospital and referred because of stricture (Group A, N = 59) or of an anastomosis dehiscence (Group B, N = 80); 97 were unrepaired and referred because of a surgical clip occluding the duct (Group C, N = 39) or bile leakage from an open duct (Group D, N = 58). All patients were surveyed in 2015 for quality of life using WHOQOL-BREF.

The mean time of follow-up was 150 months. The time without symptoms amounted to >5 years in 78.6% of patients. The mean time before anastomosis renewal ranged from 8.9 to 4.7 years (*P* < .04). Multivariate analysis showed infection, failure of reconstruction in public hospital, and female sex as factors responsible for poor long-term outcome.

Patients in Group C had better quality of life than the others (*P* < .001) with respect to physical health (median 67.85) and psychological condition (median 79.16). The overall mortality was 15.2%.

The long-term result of reconstruction depends on the cause of referral which, in turn, arises from subsequent intervention taken in local hospitals.

## 1 Introduction

The transection of the hepatic duct at or below the level of biliary bifurcation, denoted as D2 and D3 by Hannover classification, is a devastating complication of cholecystectomy.^[1]^ Restoring the bile outflow in such cases represents an extreme surgical challenge. The short-term results are generally excellent; but the long-term outcome is uncertain.^[2–12]^ This has led to much heated discussion and controversy over the timing of biliary reconstruction, treatment of complications in the post-cholecystectomy period and who ought to repair the injury and where.^[13–19]^

The aim of this study was to determine the conditions that appeared to be vital for the best outcome in patients referred to our Department from base-level hospitals with transection of the main biliary duct at the level of the liver's hilum.

## 2 Material and methods

The study involved a sample of 236 patients (M 65, F 171, median age 52.3 years) treated at the Department of General, Transplant & Liver Surgery, Medical University of Warsaw, Poland in the years 1990 to 2005. Of these, 132 had Hannover type D2 lesions and 104 Hannover type D3 lesions. Information concerning the laparoscopic procedure, the site of the ductal injury, and the presenting symptoms was obtained from operative reports, referral notes, local medical charts, and interviews with referring physicians.

**Figure 1:**
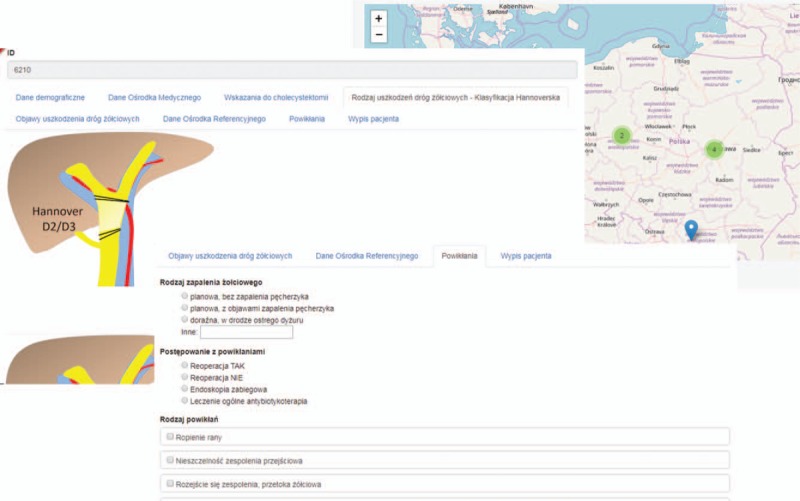
Sample forms the telemedical system, in that the spatial module (GIS).

The dataset used and described in the article consists of data collected by the telemedical system for the assessment of the adverse effects of treatment. The newly developed WEB application uses only open-source and free tools standards. It is also possible to present the analytical data using the *Geographic Information System* (GIS) solutions for the spatial presentation (Fig. 1).

### 2.1 Characteristics of patient groups

Group A consisted of 59 patients who underwent reconstruction of a damaged hepatic duct by hepaticojejunal anastomosis performed by local surgeons. The duct lesion was repaired at the time of cholecystectomy (early primary repair) in 41 patients (69.5%). In 18 patients (30.5%), the injury was recognized postoperatively within 1 to 14 days (mean 6 days) and reconstructed within 30 days (mean 17, range 1–30) after the injury (early delayed repair). Postoperative complications such as leakage of bile and intra-abdominal infection were noted in 48 patients (81.35%). All of them, however, successfully healed. They were referred because of an early stricture of the biliary anastomosis, within 6 months of initial repair. Endoscopic interventions were undertaken before referral in 23 (38.9%) of them. The time of patient referral was between 91 and 178 days (mean 115.4 days). All were eligible for reconstruction of the anastomosis after short-term medical improvement.

Group B consisted of 80 patients who underwent biliary reconstruction by hepaticojejunostomy performed by local surgeons. Intraoperative repair of the injury was made at the time of the cholecystectomy (early primary repair) in 17 patients (21.25%). In 63 patients (78.75%), the injury was recognized within 1 to 14 days and reconstructed within 30 days (mean 21, range 1–30) after the cholecystectomy (early delayed repair). The procedures failed because of a leak of the anastomosis that resulted in the intra-abdominal infection that formed within 5 to 17 days after the procedure. Fifty-five of these patients (68.5%) underwent re-laparotomy and drainage before referral. The others (31.5%) were referred without additional treatment immediately after the anastomosis leak was recognized. The time of patient referral was between 21 and 90 days (mean 42 days). After admittance, all patients required complementary treatment with broad-spectrum antibiotics, but 54 required additional drainage of subphrenic infection: 30 patients by re-laparotomy and 24 by ultrasonography guided drainage (USG)-guided drainage. Only 17 patients (21.2%) were eligible for immediate reconstruction after short-term reconstitution of medical status.

Group C consisted of 39 patients with jaundice that occurred shortly after their cholecystectomy. The course of the procedure was graded by the operative team as uncomplicated until increasing levels of bilirubin and the external symptoms of jaundice became evident. The complication was recognized after 1–7 days in 30 patients and after 7 to 14 days in 9 patients. Imaging revealed obstruction of the hepatic duct by surgical clips. The patients were not repaired at the base-level hospital but referred to our Institution within 3 to 21 days (mean 11 days) of diagnosis. All 39 patients were eligible for biliary reconstruction shortly after referral.

Group D consisted of 58 patients who were referred because of extrahepatic biloma and/or a subphrenic abscess owing to bile leakage from a hepatic duct left open. The symptoms were discovered in 1 to 7 days after the cholecystectomy in 20 patients and in 7 to 14 days in 38 patients. No attempts were made by the local teams to repair the injury, but other surgical and nonsurgical interventions were applied to control intra-abdominal infection, including re-laparotomy, drainage of the subphrenic bile collection, and broad-spectrum antibiotic therapy. The time of patient referral was between 21 and 90 days (mean 31 days). After admittance, all patients required complementary treatment with broad-spectrum antibiotics, but 44 required additional drainage of the subphrenic infection by re-laparotomy (30 patients) or USG-guided drainage (14 patients). None was eligible for immediate reconstruction.

### 2.2 Surgical management

Characteristics of the groups of patients, their management, and presentation before the definitive biliary reconstruction are provided in Table 1.

**Table 1:**
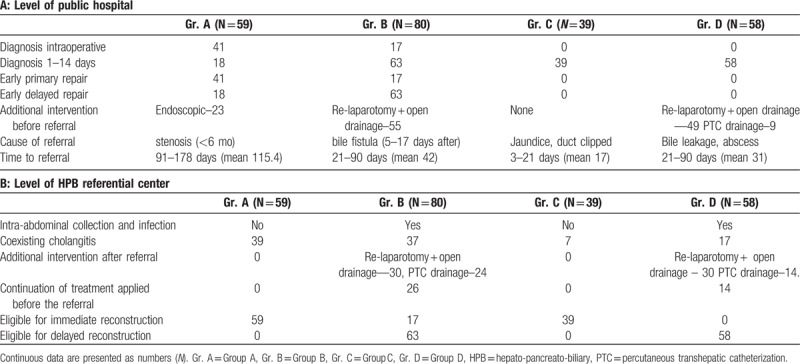
Characteristics of patient groups, management, and presentation before the definitive biliary reconstruction: A—level of public hospital; B—level of HPB referential center.

The reconstruction of the biliary tract was performed electively by the wide transvers opening of abdominal cavity. The end-to-side Roux-en-Y hepaticojejunostomy was the preferred option for the biliary reconstruction in all cases of the hepatic duct transection injury, as well as, for reconstruction in the treatment of stenotic hepatico-jejunal anastomosis. The dead tissue at the end of the duct was always debrided to secure healing of the anastomosis. In patients with transection at or above the bifurcation (Hannover D3 type), the hilar plate was mobilized to obtain an adequate length of the left hepatic duct. The stump of hepatic duct at the bifurcation was usually broadened by incision of the anterior wall of the left hepatic duct. This allowed to achieve duct section as wide as at least 1.2 to 1.5 cm, and facilitate accurate mucosa to mucosa anastomosis. The one-layer end-to-side anastomosis with 5–0 absorbable suture was carried out with the jejunal loop for bile drainage of 40 to 60 cm in length to avoid reflux and infection. The placement of 1 or 2 stenting silicone tubes into the intrahepatic ducts via the biliary-jejunal anastomosis was the standard of the procedure. Drain was placed intraoperatively at the liver site of anastomosis to prevent postoperative bilomas or intra-abdominal collections.

Cholangiography was performed routinely during the operation. The intrahepatic transjejunal tubes were connected to external drainage until days 8 to 10 after surgery, when cholangiography was again performed. If no leaks were found, the tubes were closed and left in place for a further 3 weeks and then removed. Systemic antibiotics (second/third-generation cephalosporins in combination with metronidazole) were given for 5 to 10 days.

### 2.3 The QOL 2015 survey

In 2015, 199 patients who had healed successfully took part in a survey of their quality of life. The questions concerned individual perceptions of quality of life and health. Detailed assessment of facets of all 4 domains relating to quality of life was also undertaken in all patients by means of the WHOQOL-BREF questionnaire.^[20–22]^

### 2.4 Aim of the study

The aim of the study was to evaluate the long-term effects of definitive biliary reconstruction by hepaticojejunostomy. The analysis included:

- the period of time without symptoms—categorized as <5 or >5 years);
- the need for subsequent surgical renewal of the anastomosis during 10 years of follow-up—categorized as not required, required once, or required more than once;
- the mean time to renewal of the anastomosis;
- the need for endoscopic interventions before anastomosis renewal—categorized as not required, required once, or required more than once;
- a comparison between the effects of reconstruction in groups of patients who underwent initial repair of bile-duct lesion in local departments and those who did not; and
- the quality of life of patients.

Patients with all other disruptions of the biliary system and any bile duct injury following cholecystectomy that occurred in our own Department or other Hepatopancreatobiliary Centers were excluded from the study.

## 3 Statistical analysis

The dataset was recorded on a disc and analyzed using SAS 9.4 statistical software (SAS Institute Inc., Cary, NC). Continuous variables were expressed as a mean ± SD, with a sample representativeness of 95% confidence interval. Discrete variables were presented as numbers or letters, and categorical variables were adequately labeled. The statistical analysis of mutual relations between the variables studied, as well as comparisons between groups of patients, was made using the Mann-Whitney *U* test and the analysis of variance (ANOVA) Kruskal-Wallis Test.

A multivariate analysis of logistic regression was performed to reveal the factors responsible for the efficacy of biliary repair, long-term outcome, and quality of life of patients. Independent variables adopted were: whether execution of or attempts at repair of the biliary lesion by local surgeons was immediate or delayed; causes of referral of patients to the Department; time after injury of referral of patients; the need for additional surgical or endoscopic interventions before or after referral because of intra-abdominal infection and/or subphrenic or intra-abdominal bile collection; and the age and sex of patients. Dependent variables adopted were whether or not a patient remained asymptomatic for > or <5 years after definitive biliary reconstruction; whether a patient needed surgery to renew the anastomosis during 10 years of follow-up, and how many times; mean time until renewal of the anastomosis; endoscopic interventions before surgery for the next anastomosis renewal; and the quality of life of a patient.

The reference group used in the model included patients referred to the Department without attempts at repair, patients sent shortly after the injury, patients who avoided intra-abdominal infection, and patients who required minimal treatment before definitive biliary reconstruction. A *P* value <.05 was adopted as statistically significant.

## 4 Ethics

This cohort study obtained ethical approval of the Bioethics Committee of Medical University of Warsaw as according to the law.

## 5 Results

Of the 236 patients who underwent definitive reconstruction of the biliary tract by the hepaticojejunal anastomosis, 12 (5%) died following the operation because of serious biliary and general complications. Biliary reconstruction also resulted in minor surgical complications, such as wound infection, bile leakage around stents, and the prolapse of biliary stents. This occurred in 27 (11.44%) patients.

Follow-up observation of the 224 patients who healed successfully was undertaken at regular intervals of 12 months until 2015. During this period, 23 (10.26%) patients died: 8 owing to secondary biliary cirrhosis and liver insufficiency and 15 owing to advanced age, circulatory insufficiency, and other general reasons. One hundred ninety-nine (84.3%) patients took part in a survey of quality of life in 2015. The mean time of follow-up for patients in Groups A, B, C, and D was respectively 153 months (range 11–283 months), 144 months (range 23–228), 155 months (range 12–291), and 142 months (range 9–241). Details are presented in Table 2.

**Table 2:**
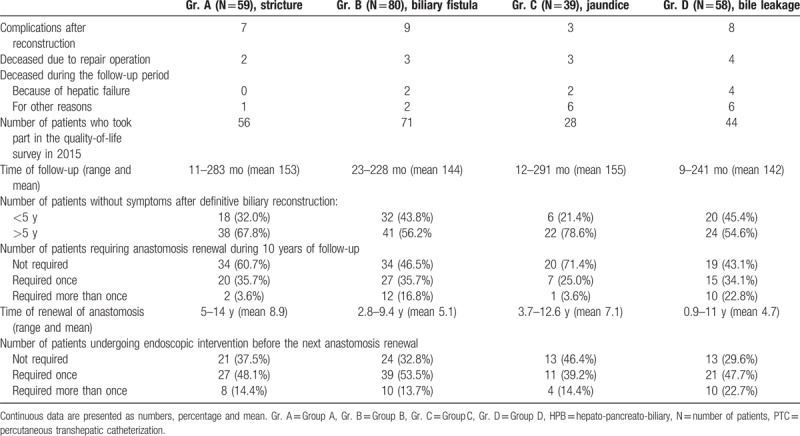
Characteristics of the patient groups, management and presentation after definitive biliary reconstruction.

### 5.1 Long-term outcome following definitive biliary reconstruction

The time without symptoms after definitive reconstruction, indicating effectiveness of the biliary anastomosis, was >5 years in 78.6% of patients from Group C, but only 67.8% of patients from Group A, 56.2% of patients from Group B, and 54.6% of patients from Group D. The indicated difference between the groups was statistically significant (*χ*^2^ = 15.61, *P* < .001). The results of multivariate analysis showed intra-abdominal infection and the subphrenic bile collection that developed after the initial operation; failure of local surgeons in reconstructing the bile duct; symptoms of cholangitis in and before definitive biliary reconstruction and female sex were independent factors that adversely influenced the length of time without symptoms following the reconstruction in our Department. Details are presented in Table 3.

**Table 3:**
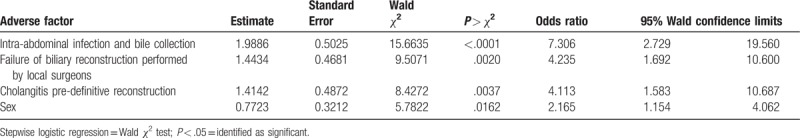
Adverse factors influencing the time without symptoms after definitive biliary reconstruction, identified by multivariate analysis with stepwise logistic regression.

The long-term effects of definitive biliary reconstruction were satisfactory in >71% of patients from Group C and 60% of patients from Group A. Most of them remained asymptomatic, but 25% and 35% of patients from Groups C and A respectively underwent surgical renewal of the anastomosis within 10 years, and 3.6% of the patients from Groups C and A more than once. The long-term effects were much worse in patients from Groups B and D wherein <50% were asymptomatic, and 16% of patients in Group B and 22% of patients in Group D underwent surgical renewal of the anastomosis more than once within 10 years (as shown in Table 2). The time until the anastomosis was renewed ranged between 5 and 14 years (mean 8.9) and 3.7 and 12.6 years (mean 7.1) for patients in Groups A and C, respectively, but between 2.8 and 9.4 years (mean 5.1) and 0.9 and 11 years (mean 4.7) for the patients in Groups B and D, respectively. The differences between the groups were statistically significant (*χ*^2^ = 4.09, *P* < .04). The results of multivariate analysis showed intra-abdominal infection and sub-phrenic bile collection that developed after the initial operation and delay in the execution of repair of the biliary lesion by local surgeons were significant factors. Details are presented in Table 4.

**Table 4:**
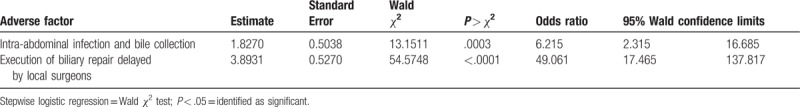
Adverse factors influencing the need for surgical renewal of the anastomosis within 10 years after definitive biliary reconstruction, identified by multivariate analysis with stepwise logistic regression.

### 5.2 The quality-of-life assessment

The median score for quality of life of 199 (84.7%) patients who underwent definitive reconstruction of the bile duct and were surveyed in 2015 using the WHOQOL-BREF questionnaire was >60 transformed points (min: 16 to max: 95) across all 4 domains. Details are presented in Table 5.

**Table 5:**
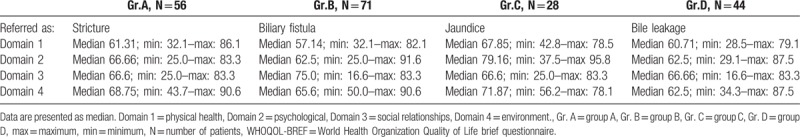
Descriptive statistics for selected quantitative variables in the study groups of the 199 patients who underwent biliary reconstruction took part in the WHOQOL-BREF quality-of-life assessment in 2015. The data are expressed as the amount of transformed points of each group of patients.

**Figure 2:**
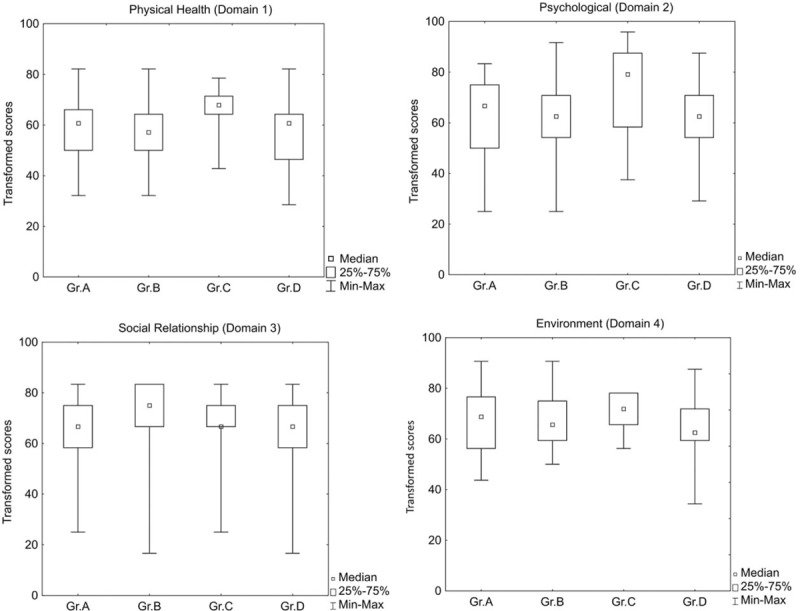
Scores indicating the quality of life of the 199 patients after at least 10 years of follow-up after definitive biliary reconstruction, surveyed in 2015 according to WHOQOL-BREF questionnaire.

The patients of Group C, reconstructed after post-cholecystectomy jaundice caused by occlusion of the hepatic duct by a surgical clip, felt they had a better quality of life in terms of physical health (median 67.85, min: 42.8 to max: 78.5) and psychological condition (median 79.16, min: 37.5 to max: 95.3) when compared to patients from the other groups. The ANOVA test showed statistically significant differences in both domains: physical health (H = 15.28, *P* < .001) and psychological condition (H = 13.19, *P* < .004). The median scores of quality of life, however, in terms of the patients’ social functioning (Domain 3) and environment (Domain 4) were remarkably similar in all groups (median 63–75, min: 16.6 to max: 95.8 of transformed points). This difference was not statistically significant. Details are presented in Figure 2.

Finally, the ANOVA test showed a significant difference overall for long-term outcome between patients of Group C and all other groups of patients. Details are presented in Table 6.

**Table 6:**
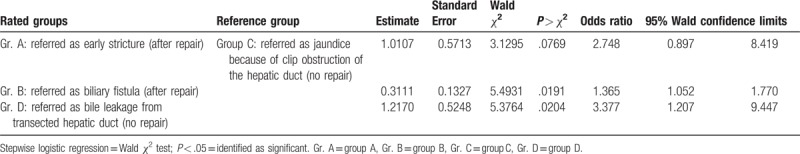
The results of multivariate analysis of the differences between the patient groups established based on causes of their referral for definitive biliary reconstruction.

## 6 Discussion

The injuries were recognized during the cholecystectomy operation in 41.7% of our 236 biliary reconstruction patients. Similar rates are reported by other data centers.^[5,9,10,17,23]^ Data available in the Medicare database indicated that almost 70% of primary care surgeons undertake repairing the injury themselves.^[9,14,17,19]^ Iannelli et al (2013) estimate the frequency of immediate repair at 35.7%,^[24]^ and Sicklick et al (2005) at 40%.^[9]^ In contrast, the rate of intraoperatively unrecognized injuries of the biliary system ranges from 35% to 65% of cases, depending on the analyzed series. Diagnosis is made upon re-laparotomy owing to peritonitis and a leak of bile from the transected lumen of the hepatic duct.^[23–28]^ Therefore, more than half the patients are referred with post-cholecystectomy intra-abdominal inflammatory complications, 58% in our case. They were referred usually between 21 and 90 days (mean, 31 days). Those who developed jaundice because of a clip-obstructed hepatic duct were typically referred sooner in our patients, before 21 days (mean, 17 days). Many reports, however, indicate patients with postoperative jaundice are referred later than those with a bile leak and intra-abdominal inflammatory complications.^[5,7,8,14,24,28]^ After reconstruction, all our patients remained under the medical supervision of our outpatient department for at least 10 years, in many cases for >24 years.

The study indicates how the level of efficacy of a definitive repair by experienced specialists is dependent upon the causes of referral, meaning the clinical patient's condition, and the gravity of the post-cholecystectomy complications. The outcomes of our reconstruction were best for patients who had not been repaired by surgeons at public hospitals and for those admitted because of the occlusion of the duct by a clip. The results were also positive in patients who successfully underwent repair at a public hospital but whose anastomosis narrowed soon afterwards. Most of these patients remained asymptomatic for at least 10 years after definitive repair, and only 3.6% of them required surgical renewal of the anastomosis more than once within 10 years. Undoubtedly, lack of intra-abdominal infection before and at the time of reconstruction was highly beneficial for a successful outcome. The outcome in all patients that presented with intra-abdominal infection, bile collection, and sepsis was significantly worse with respect to all points of study interest, and they experienced shorter duration of the asymptomatic period between renewals of the anastomosis, and shorter periods of time between subsequent anastomosis renewal. de Reuver et al (2008) listed extended injury to the bile duct and acute repair as independent negative predictors on outcome^[29]^; Schmidt et al (2004) indicated the presence of combined bile duct and hepatic artery injury as the cause of poor outcome;^[18]^ Stilling et al (2014) found in Danish national study a considerable risk of long-term complication in the bile duct injury repaired by early hepatico-jejunostomy,^[5]^ as factors that causes unsatisfactory outcome of repair. Our study indicates the failure of reconstruction undertaken in public hospitals, especially if the execution of repair was delayed by surgeons who do not specialize in hepatopancreatobiliary surgery, as well as, intra-abdominal infection after the initial operation, the onset of cholangitis before the reconstruction, and being female as the most important factors adversely influencing the length of time without symptoms and the time until renewal of the anastomosis.

Many observations demonstrate a significant long-term reduction both in physical and mental quality of life after bile duct injury, even if the injury was successfully repaired.^[14,27,24,30–37]^ Using the WHOQOL-BREF questionnaire, the median score of 199 patients (84.7%) who underwent the definitive biliary reconstruction in our Institution was >60 transformed points (min:16 to max: 95) with respect to all 4 domains. Surprisingly, the median score with respect to the social functioning of patients (Domain 3) and environment (Domain 4) was remarkably similar between all groups, which indicates the social and economic alignment of patients undergoing cholecystectomy. The patients reconstructed because of occlusion of the hepatic duct (Group C) evaluated their quality of life much better with respect to Domain 1 and Domain 2 than patients from the other groups. Also, the quality of life of patients from Group A seemed satisfactory. However, the quality of life of patients who suffered bile leakage and subphrenic infection after the initial operation (Group B, D) was significantly worse with respect to both physical health and psychological conditions. The general status, starting point, and degree of preparation of these patients for final biliary reconstruction were more demanding from a medical and surgical point of view. The results indicated that both the final result and patient satisfaction, omitting of all other determinants, depended on the causes of referral for biliary reconstruction. These, in turn, arise from subsequent interventions taken by staff in base-level hospitals. This finding differs from those reported by Sarmiento et al (2004) and Hogan et al (2009) who found no effect of bile duct injury on the physical and mental quality of life of patients.^[31,36]^ Our study's findings are consistent with those of de Reuver et al,^[29]^ Melton et al,^[30]^ Landman et al,^[31]^ and Boerma et al,^[36]^ who all reported detriment in most domains of a patient's life after major bile duct injury.

Certainly, this analysis may exhibit some bias characteristic of studies carried out on a large sample of patients with a very specific type of injury. The criteria used to recruit and enroll patients into separate study groups were basically the same. The cross-reference of data by patient and by surgeon in charge at the time of admission was saved in the database. All questionable cases were excluded from further analysis. All procedures were performed by the same highly experienced specialists, according to the same operative procedures. The renewals of anastomosis were undertaken by the surgeons who carried out the definitive repair previously. We believe any variations in performance of the procedure caused by the individual nature of a particular surgeon were too small to generate significant differences. Nevertheless, the cause–effect relation between procedures and a patient's long-term outcome may have given rise to a small amount of bias in interpretation of the results. Another potential source of bias may also originate from estimation of quality of life by the patients themselves. This is a phenomenon similar to and recognized in quality-of-life assessments in patients with cancer (denoted as “response-shift bias”), who report better quality of life than expected.^[27,38]^ Patients with bile duct injury typically report worse evaluations of their quality of life, as most remain annoyed because of injury, prolonged hospital stay, additional invasive interventions, and, most of all, doubts about the future. Also, the fact that this was a single-center analysis may itself constitute a limitation of the study.

It is recommended that reconstruction of a bile duct injured at the hilum of the liver should be in specialized hepatobiliary centers.^[1,9,23,28,31,32,38–41]^ Despite strenuous attempts, this recommendation is far from being implemented in reality. Reports from many centers indicate an upward trend in repair attempts by general surgeons in the case of simple and complicated injuries. In view of these facts, advanced training for general surgeons in proper surgical skills is recommended.

## 7 Conclusions

1. The result of reconstruction depends on the causes of referral that, in turn, arise from subsequent intervention taken in the public hospitals;
2. Proper training of general surgeons in nontechnical and surgical skills may reduce the frequency both of injury and of failed bile duct repairs;
3. Failure of reconstruction in public hospitals, intra-abdominal infection, cholangitis, and being female should be considered as negative independent factors influencing the need for the hepaticojejunal anastomosis renewal and the length of time after surgery when patients are asymptomatic;
4. Satisfactory long-term outcomes and an acceptable level of quality of life with respect to all 4 Domains indicated in the WHOQOL-BREF questionnaire were achieved after the definitive biliary reconstruction in our sample of 199 patients.

## Acknowledgments

The authors thank the team at the Department of General, Transplant, and Liver Surgery, Medical University of Warsaw for their tremendous contribution to the treatment of complex cases, such as the iatrogenic injury of the bile ducts.

## Author contributions

**Conceptualization:** Włodzimierz Otto.

**Data curation:** Włodzimierz Otto, Justyna Smaga, Krzysztof Dudek.

**Formal analysis:** Janusz Sierdzinski, Włodzimierz Otto.

**Investigation:** Włodzimierz Otto, Justyna Smaga, Janusz Sierdzinski.

**Methodology:** Wlodzimierz Otto, Justyna Smaga, Janusz Sierdzinski.

**Project administration:** Wlodzimierz Otto, Krzysztof Zieniewicz, Krzysztof Dudek.

**Software:** Janusz Sierdzinski.

**Supervision:** Włodzimierz Otto, Krzysztof Zieniewicz.

**Validation:** Janusz Sierdzinski, Włodzimierz Otto, Krzysztof Zieniewicz.

**Visualization:** Janusz Sierdzinski, Justyna Smaga.

**Writing – original draft:** Włodzimierz Otto, Janusz Sierdzinski.

**Writing – review & editing:** Krzysztof Zieniewicz.
